# Biochemical Markers Predictive of Preterm Delivery

**DOI:** 10.1155/S1064744997000240

**Published:** 1997

**Authors:** Steven R. Inglis

**Affiliations:** 1 Division of Maternal-Fetal Medicine Department of Obstetrics and Gynecology New York Hospital-Cornell Medical Center 525 E. 68th St. J-130 New York New York 10021 USA; 2 Division of Maternal-Fetal Medicine Department of Obstetrics and Gynecology Lincoln Medical and Mental Health Center Bronx New York USA

## Abstract

Preterm delivery is the leading cause of perinatal morbidity and mortality worldwide. Despite a great deal of research into this disease, we still do not understand its pathophysiology. Our treatments for this disease are only marginally effective. Biochemical markers were developed with the hope of giving us new tools to prevent preterm deliveries. Specifically the hope was that they could predict which patients were destined to have a preterm delivery. At the present time these markers perform only satisfactorily at predicting preterm labor. They are expensive and not convenient to use at present. Perhaps more importantly, though, these markers have given us insight into the complexities of preterm delivery. Preterm delivery can arise from many different etiologies. This will lead to research into new treatments as knowledge about preterm delivery is amassed. We know that any number of pathological processes may be involved in any given patient with preterm labor. Biochemical markers have the distinct advantage of being able to determine the specific pathophysiology in a given patient and may allow us to tailor therapy according to the specific problem. In the future it is likely that a careful search for specific pathophysiology will be the only way we can treat this disease effectively. For the present time the biochemical markers will be used only to predict preterm delivery. Ultrasound measurements of the cervix during the pregnancy are likely a faster and less expensive way to accomplish that goal.


Infectious Diseases in Obstetrics and Gynecology 5:158–164 (1997)
© 1997 Wiley-Liss, Inc.

### Biochemical Markers Predictive of Preterm Delivery

**Steven R. Inglis**

*Division of Maternal-Fetal Medicine, Department of Obstetrics and Gynecology, New York Hospital-Cornell Medical Center, New York, New York; Division of Maternal-Fetal Medicine, Department of Obstetrics and Gynecology, Lincoln Medical and Mental Health Center, Bronx, New York*

ABSTRACT

**Preterm delivery is the leading cause of perinatal morbidity and mortality worldwide. Despite a great deal of research into this disease, we still do not understand its pathophysiology. Our treatments for this disease are only marginally effective. Biochemical markers were developed with the hope of giving us new tools to prevent preterm deliveries. Specifically the hope was that they could predict which patients were destined to have a preterm delivery. At the present time these markers perform only satisfactorily at predicting preterm labor. They are expensive and not convenient to use at present. Perhaps more importantly, though, these markers have given us insight into the complexities of preterm delivery. Preterm delivery can arise from many different etiologies. This will lead to research into new treatments as knowledge about preterm delivery is amassed. We know that any number of pathological processes may be involved in any given patient with preterm labor. Biochemical markers have the distinct advantage of being able to determine the specific pathophysiology in a given patient and may allow us to tailor therapy according to the specific problem. In the future it is likely that a careful search for specific pathophysiology will be the only way we can treat this disease effectively. For the present time the the biochemical markers will be used only to predict preterm delivery. Ultrasound measurements of the cervix during the pregnancy are likely a faster and less expensive way to accomplish that goal. Infect. Dis. Obstet. Gynecol. 5:158–164, 1997.** © 1997 Wiley-Liss, Inc.

KEY WORDS

Pregnancy, preterm delivery, biochemical markers, ultrasound

Despite advances in obstetrical and perinatal care during the past several decades, the rate of preterm birth has remained unchanged or has risen.[1,2] Specifically, the extensive use of tocolytic agents has done little to reduce perinatal morbidity and mortality from preterm delivery.[3,4] Despite its pivotal role in management, identification of patients at risk for preterm delivery is difficult. Current methods of detecting patients at risk for preterm delivery rely on obstetric history, demographic factors, or premonitory symptoms that are neither sensitive nor specific.[5] Biochemical markers were developed in the hope that they could predict which patients were destined to have a preterm delivery at an early enough interval such that the delivery could be avoided. Biochemical markers can be categorized into one of a number groups. The first group is the inflammatory mediators, or cytokines, which are produced during preterm labor in response to infection. Fetal fibronectin (FFN) is a glycoprotein produced by the fetus at the interface of the placenta and membranes with the uterine decidua during pregnancy. It can frequently be identified in the lower genital tract secretions in very early pregnancy or late pregnancy. However, in the early third trimester its presence is

Correspondence to: Dr. Steven R. Inglis, New York Hospital-Cornell Medical Center, 525 E. 68th St. J-130, New York, N.Y. 10021.

Received 1 October 1997

Accepted 21 October 1997

associated with a high risk of preterm delivery. It is not clear whether an inflammatory process is responsible for its production or release into the lower genital tract. Enzymes such as metalloproteinase, phospholipase, sialidase, and protease measured in the blood or lower genital tract during pregnancy have been associated with preterm delivery. Also endocrinological markers such as estriol are being studied intensively and may present a new approach to predicting preterm delivery.

### CYTOKINES

The chemical messengers which we produce in response to an infection or tissue injury are called cytokines. These messengers communicate to the immune system that an infection is present and initiate a response. Later they modulate the response during the healing process. It is estimated that at least 20% of premature neonates are born to mothers with intraamniotic infection.[6] In the presence of intraamniotic infection, markedly elevated levels of inflammatory mediators, or cytokines, are seen in the amniotic fluid. The cytokines interleukin-1, interleukin-6, and tumor necrosis factor-α have all been measured in the amniotic fluid.[7–10] The inflammatory mediators then stimulate prostaglandin synthesis by a variety of uterine tissues. The prostaglandins then lead to parturition.[11–13] Also, elevated cytokine levels in the amniotic fluid predict preterm labor that is refractory to tocolysis resulting in preterm delivery.[13] Elevated levels of cytokines measured non-invasively from the lower genital tract also predict preterm delivery.[14,15]

Interleukin-6 is a major mediator of the host response to infection and tissue injury. It is normally present at low levels in human amniotic fluid in the second and third trimesters. Spontaneous labor at term is associated with a modest increase in the level of interleukin-6.[12] Intraamniotic infections, however, are associated with a dramatic increase in amniotic fluid interleukin-6 levels.[12,13]

Tumor necrosis factor-α is a cytokine produced by activated macrophages in response to a variety of stimuli, including bacterial products, viruses, and parasites. Endotoxin (lipopolysaccharide), a component of the cell wall of gram-negative bacteria, can itself stimulate decidual cells to produce tumor necrosis factor.[16] No detectable tumor necrosis factor-α was found in the amniotic fluids of women in the second trimester, third trimester, or during normal spontaneous labor.[10,17] Once again, in the setting of microbial invasion of the amniotic cavity, high levels of tumor necrosis factor-α are found in the amniotic fluid.[13] In our center we found that among patients admitted to the hospital with threatened preterm labor, women found to have tumor necrosis factor-α in the lower genital tract had a greater than six-fold increased risk of preterm birth.

A new and concerning development is the association of inflammation at the time of parturition and increased risk of neurological problems. One study showed an association between multiple cytokines found in the amniotic fluid during preterm delivery, neonatal brain white matter lesions, and cerebral palsy.[18] Another study found that exposure of a term infant to intrauterine infection such as fever or a clinical diagnosis of chorioamnionitis was associated with a 9 times higher risk of cerebral palsy.[19] These findings in both term and preterm infants only increase the importance of further study and understanding of preterm delivery.

### FETAL FIBRONECTIN

Fibronectins are large glycoproteins that bind cells to the extracellular matrix.[20] They are a family of ubiquitous proteins found in the plasma and extracellular matrix. Although similar in structure to two other isoforms of this protein, FFN is distinguished immunochemically by a unique epitope resulting from alternate splicing of the primary messenger RNA transcript. Fetal fibronectin is specific to the fetus and trophoblast. High concentrations are found in the amniotic fluid. Immunohistochemical studies demonstrated FFN throughout the chorion layer of the fetal membranes, and between the uterine decidua and intervillous space and the cytotrophoblastic cell columns.[20]

Fetal fibronectin detected in cervical and vaginal secretions during the second and third trimester have been associated with an increased risk of preterm delivery.[20,21] In our study we found that women positive for FFN during threatened preterm labor had almost a five-fold increased risk for preterm birth ($p < 0.05$).[14] Since FFN is normally present in amniotic fluid and placental tissue, its appearance in the lower genital tract is suggestive of mechanical leakage or inflammation-mediated damage to the integrity of the membranes.[20] Cross-sectional studies which examined women with symptoms of preterm labor or rupture of membranes and longitudinal prospective cohort studies of women at low risk report that the presence of vaginal or cervical FFN is associated with a four-fold to nine-fold increased risk of preterm delivery.[22,23] Preliminary information shows that FFN can be recovered from cervical fluid approximately 3 weeks prior to the onset of preterm labor or preterm rupture of membranes.[22]

Alternatively, the FFN could be released or produced in large amounts secondary to inflammation in the chorion, decidua, or cervix. We found an independent association between inflammatory markers and FFN.[19] The cytokines may induce the production of FFN in the fetal membranes or decidua. Interestingly, fibronectin is susceptible to proteolytic enzyme activity by both bacteria and inflammatory cells.[24] Release of proteolytic enzymes, either by bacteria or during the processes of inflammation, could lead to the destruction of FFN and disruption of the chorion-decidua interface. Documentation of associations between specific reproductive tract infections, virulence factors, or placental villitis and the presence of FFN would provide support the idea that infection contributes to the release of FFN. Identification of a specific marker for infection-mediated preterm birth may allow for treatment of the infection and its inflammatory responses.

### ELASTASE, PROTEASE, PHOSPHOLIPASE

The cervix is composed primarily of collagen and therefore vulnerable to the effects of bacterial and/ or host-mediated proteolytic enzymes. The action of exogenous proteases and collagenase on a cervix could accelerate the processes towards preterm birth. Microorganisms produce a variety of proteolytic enzymes, including collagenase, elastases, IgA protease, sialidase, and mucinases. These enzymes may be involved both in overcoming mucous membrane of the vagina, mucous plug, and other endocervical host defenses and weakening the fetal membranes.[25–28] Sialidase can break down mucin and facilitate bacterial attachment (the first step in establishing bacterial infection). These changes can lead to disease progression with spread of infection up into the uterus.

Similarly, proteases may act as immunogenic agents by activating host inflammatory responses. Release of inflammatory mediators then leads to prostaglandin activation. Alternately, the proteolytic enzymes may directly break down cervical collagen and the amniochorion. This then leads to premature cervical ripening, weakening of fetal membranes, and premature rupture of the membranes.[32] Phospholipase $A_2$ ($PLA_2$) may directly disrupt collagen biosynthesis and participate in cervical ripening and weakening of the fetal membranes.[25] $PLA_2$ action may involve direct release of arachidonic acid and subsequent prostaglandin synthesis by maternal tissues.[25]

Vaginal fluid levels of sialidase, $PLA_2$, prostaglandin $E_2$, and interleukin 1-beta are greatly increased among women with bacterial vaginosis.[25,28] The role of these putative virulence factors during pregnancy has not been studied in women. However, such microbe-produced substances (i.e., collagenase) along with similar enzymes produced of such virulence factors within the vagina may predispose to premature cervical ripening, preterm labor, or premature rupture of membranes. An association between virulence-factor presence and shortened cervical length or funneling would support these processes.

### PROLACTIN

Prolactin is produced by the decidua, maternal adenohypophysis, and fetal pituitary. Decidual production of prolactin is induced by the alpha subunit of human chorionic gonadotropin. Prolactin is then diffused across the membranes to the amniotic cavity. The role of the prolactin is not clear, but it may suppress the synthesis of prostaglandins and augment fetal lung maturity. High levels of prolactin are found in the amniotic fluid during the second and third trimesters. Prolactin measured in the washings of the ectocervix and vaginal fornices predicts those patients likely to deliver at 34 weeks of gestation or before, as well as those with shorter latency to delivery and deliveries with lower birthweights.[29] The question of whether prolactin is actively produced and shed through the cervix or leaked subclinically through the fetal membranes remains unresolved.

### ESTRIOL

Considerable information suggests that the fetal and maternal endocrine systems are involved in the biology of both term and preterm parturition in mammalian species. For humans, the association between the levels of estrogen hormones in blood and delivery is less clear. Unlike hormone levels found in serum or urine, salivary fluid levels reflect only the unbound or free fraction of the hormone.[30] For this reason there has been interest in using salivary estriol ($E_3$) levels to predict delivery. More than 90% of $E_3$ is derived from fetal sources and its production increases approximately 3 weeks prior to the onset of term labor.[31] It appears that labor does not occur spontaneously post-term (after 42 weeks of gestation) without this rise in $E_3$.[32] New information has shown that salivary $E_3$ concentrations increase prior to spontaneous preterm labor in approximately 80% of cases. Increases in $E_3$ have been associated with induction of many uterine changes. For example, formation of oxytocin receptors, gap-junction proteins, and prostaglandin synthesis have been reported.

### METALLOPROTEINASE

Metalloproteinases are a family of enzymes including collagenase, gelatinase, and stromelysins. These enzymes are produced by the body to aid in the breakdown of tissues. There are naturally occurring inhibitors of these enzymes called tissue inhibitors of metalloproteinases. During pregnancy, high levels of this inhibitor are found in the amniotic fluid. On the other hand, the serum levels are lower than in the nonpregnant state.[33] Most importantly, serum levels of the inhibitor increase significantly prior to both term and preterm labor states.[33]

### ELEVATED MATERNAL SERUM ALPHA-FETOPROTEIN

Screening all pregnant women for the maternal serum level of alpha-fetoprotein (MSAFP) is well established to help detect the presence of neural tube defects and aneuploidy.[34] Approximately 2–3% of all women screened have elevated MSAFP levels which can not be explained by correction of gestational age assessment, presence of multiple gestations, fetal death, or presence of an anomalous fetus.[34] Increasing information suggests that high levels of MSAFP in the presence of a structurally normal fetus is associated with increased risk for multiple adverse pregnancy outcomes. Among these studies, a two-fold to ten-fold increase in risk for preterm birth associated with unexplained elevated MSAFP has been noted.[34–36] Preterm rupture of membranes, intrauterine growth retardation, stillbirth, pregnancy-induced hypertension, and placental abruption are also increased significantly.[34–36] Why elevated MSAFP is related to preterm birth is unclear. It is thought that alpha-fetoprotein is produced in the fetal liver and enters the maternal serum by crossing the placenta or through diffusion across the amniochorion.[35] Presence of an abnormally high maternal serum level may reflect placental dysfunction or damage to the maternal-placental barrier.[37] Evidence at delivery of either placental inflammation (villitis) or old thrombosis has been demonstrated among up to 70% of women who had an elevated MSAFP in the second trimester.[38] The etiology of such early placental damage remain unclear; however, chronic infection and inflammation of the uterine lining (decidua) or acute ascending infection may play a role.

### CERVICAL SONOGRAPHY AND PRETERM DELIVERY

Ultrasound evaluation of the cervix is being used increasingly to assist in the management of preterm labor and as a screen to identify patients at high risk for preterm delivery. We understand that data obtained by measuring the cervix with the tranabdominal approach is far less reliable than that obtained from the transvaginal approach. Transvaginal ultrasound of the cervix is being used in three clinical settings in regard to preterm delivery. It is being used during the management of women with suspected cervical incompetence, in screening programs of women at high risk for evidence of cervical shortening, and during evaluation of preterm labor.[39–41] Transvaginal ultrasound offers an objective method to evaluate the cervix of a woman with suspected cervical incompetence. For women with unclear histories for cervical incompetence, such as second trimester losses with painful dilation, premature rupture of membranes in the second trimester, or a short cervix on digital exam, transvaginal ultrasound of the cervix adds valuable data to help manage the patient. It can assist in the decision regarding cerclage placement. The cervical length should remain more than 3 centimeters between 14 and 20 weeks. If the length is between 2.5 and 3 centimeters, more careful monitoring is indicated. Patients with questionable histories of cervical incompetence and a cervical length less than 2.5 centimeters may benefit from cerclage. This recommendation is based on the risk of rapid cervical shortening with loss of the fetus. Studies of transvaginal ultrasound of the cervix for cervical length in pregnancy have established norms. Most studies show that between 10 and 30 weeks gestation, a cervical length of less than 2.5 centimeters occurs in less than the tenth percentile.[41] Moreover, a short cervix predicts a high risk of preterm delivery. Women with a history of multiple preterm deliveries or multiple gestations may benefit from monitoring the cervical length during pregnancy. Thus far transvaginal ultrasound of the cervix compares favorably with the biochemical markers in its ability to predict preterm delivery.[42]

### THE FUTURE OF BIOCHEMICAL MARKERS

It is clear that the pathophysiology of spontaneous preterm delivery is poorly understood. It is not likely that either biochemical markers or cervical sonography will perfectly predict preterm delivery. Inflammatory markers and FFN are occasionally going to be present in the lower genital tracts of women during uneventful pregnancies. When they are detected in the presence of uterine contractions, there is a much higher liklihood of preterm delivery. Localized, immune-system activation undoubtedly takes place in the cervix to prevent vaginal microorganisms from ascending to the uterus. The initiation and extent of cytokine production in response to various stimuli is under genetic control and would be expected to vary between individuals.[43]

None of the biochemical markers has turned out to be the "solution" to preterm delivery. Substantive improvements in our clinical management and outcome of preterm labor can only come with understanding of its pathophysiology. These markers allow us to objectively determine pathophysiology in at least some of the mothers who have a preterm delivery. Only through investigations of the biochemical markers can we begin to understand the complex changes that occur during a preterm delivery. Once we understand the pathophysiology of preterm delivery, we can then set about finding the elusive solution to the problem of preterm delivery. The biochemical markers may well prove useful in the future to assist us in tailoring therapy for individual patients with specific problems that are amenable to medical or surgical therapy. As seen with the recent findings of increased risk of neurological disease with evidence of inflammation, they may spur us to use new therapies or, in some cases, assist in an expedient delivery. In the meantime, cervical sonography does have appeal because it simply looks for an anatomic defect. It is non-invasive, relatively inexpensive, and predictive of preterm delivery in many cases. It appears that transvaginal ultrasound of the cervix will perform as well as any of the biochemical markers in predicting preterm delivery. It has an important role in the management of cervical incompetence and preterm labor. Ultimately, like the biochemical markers, even the usefulness of cervical sonography will be limited until we understand the pathogenesis of preterm delivery.

### REFERENCES

1. Committee to Study the Prevention of Low Birth Weight: Division of Health Promotion and Disease, Institute of Medicine: Preventing low birth weight. Washington, D.C.: National Academy Press, 1985.
2. Creasy RK: Preterm birth prevention: Where are we? Am J Obstet Gynecol 168:1223–1230, 1993.
3. Boylan P, O'Driscoll K: Improvement in the perinatal mortality rate attributed to spontaneous preterm labor without use of tocolytic agents. Am J Obstet Gynecol 145:781–783, 1983.
4. The Canadian Preterm Labor Investigators Group: Treatment of preterm labor with the beta-adrenergic agonist ritodrine. N Engl J Med 327:308–312, 1992.
5. Main DM, Gabbe SG, Richardson D, Strong S: Can preterm deliveries be prevented? Am J Obstet Gynecol 151:892–898, 1985.
6. Romero R, Mazor M, Wheyey YK, et al.: Infection in the pathogenesis of preterm labor. Semin Perinatol 12:262–279, 1988.
7. Romero R, Mazor M, Brandt F, et al.: Interleukin-1α and interleukin-1β in preterm and term human parturition. Am J Reprod Immunol 27:117–123, 1992.
8. Romero R, Avila C, Santhanam U, Sehgal P. Amniotic fluid interleukin-6 in preterm labor. Association with infection. J Clin Invest 1990;85:1392–1400.

9. Romero R, Mazor M, Sepulveda W, Avila C, Copeland D, Williams J: Tumor necrosis factor-α in preterm and term labor. Am J Obstet Gynecol 166:1576-1587, 1992.

10. Hillier SL, Witkin SS, Krohn MA, Watts DH, Kiviat ND, Eschenbach DA: The relationship of amniotic fluid cytokines and preterm delivery, amniotic fluid infection, histologic chorioamnionitis, and chorioamnion infection. Obstet Gynecol 81:941–948, 1993.

11. Novy MJ, Liggins GC: Role of prostaglandins, prostacyclin and thromboxanes in the physiologic control of the uterus and in parturition. Semin Perinatol 4:45–66, 1980.

12. Romero R, Durum S, Dinarello CA, Oyarzun E, Hobbins JC, Mitchell MD: Interleukin-1 stimulates prostaglandin biosynthesis by human amnion. Prostaglandins 37:13–22, 1989.

13. Romero R, Sepulveda W, Kenney JS, Archer LE, Allison AC, Sehgal PB: Interleukin 6 determination in the detection of microbial invasion of the amniotic cavity. Ciba Found Symp 167:205–220, 1992.

14. Inglis S, Jeremias J, Kuno K, et al.: Detection of tumor necrosis factor-α, interleukin-6, and fetal fibronectin in the lower genital tract during pregnancy: Relation to outcome. Am J Obstet Gynecol 171:5–10, 1994.

15. Lockwood CJ, Ghidini A, Wein R, Lapinski R, Casal D, Berkowitz RL: Increased interleukin-6 concentrations in cervical secretions are associated with preterm delivery. Am J Obstet Gynecol. 171:1097–1102, 1994.

16. Casey ML, Cox SM, Beutler B, Milewich L, MacDonald PC: Cachectin/tumor necrosis factor-α formation in human decidua. Potential role of cytokines in infection-induced preterm labor. J Clin Invest 83:430–436, 1989.

17. Romero R, Mazor M, Sepulveda W, Avila C, Copeland D, Williams J: Tumor necrosis factor-α in preterm and term labor. Am J Obstet Gynecol 166:1576–1587, 1992.

18. Yoon BH, Jun JK, Romero R, et.al.: Amniotic fluid inflammatory cytokines (interleukin-6, interleukin-1β, and tumor necrosis factor-α), neonatal brain white matter lesions, and cerebral palsy. Am J Obstet Gynecol 177:19–26, 1997.

19. Grether JK, Nelson KB: Maternal infection and cerebral palsy in infants of normal birth weight. JAMA 278:207–211, 1997

20. Lockwood CJ, Senyei AE, Dische MR, et al.: Fetal fibronectin in cervical and vaginal secretions as a predictor of preterm delivery. N Engl J Med 325:66–74, 1991.

21. Eriksen NL, Parisi VM, Daoust S, Flamm B, Garite TJ, Cox SM. Fetal fibronectin: A Method of detecting the presence of amniotic fluid. Obstet Gynecol 80:451–454, 1992.

22. Lockwood CJ, Senyei AE, Desche MR, et al.: Fetal fibronectin in cervical and vaginal secretions as a predictor of preterm delivery. N Engl J Med 325:669–674, 1991.

23. Lockwood CJ, Wein R, Lapinski R, et al.: The presence of cervical and vaginal fetal fibronectin predicts preterm delivery in an inner-city obstetric population. Am J Obstet Gynecol 169:798–804, 1993.

24. Draper D. McGregor JA: Protease activity of *Trichomonas vaginalis* cleaves fetal fibronectin and limits the ability to detect the molecule. Presented at Infectious Diseases Society for Obstetrics and Gynecology Monterey, CA August 3–6, 1994.

25. McGregor JA, French JI, Jones W, et al.: Associations of cervico/vaginal infections with increased vaginal fluid phospholipase $A_2$ activity. Am J Obstet Gynecol 167: 1588–1594, 1992.

26. McGregor JA, Lawellin D, Franech JI, et al.: Bacterial vaginosis is associated with prematurity and vaginal fluid mucinase and sialidase: Results of a controlled trial of topical clindamycin cream. Am J Obstet Gynecol 170: 1048–1060, 1994.

27. Schoonmaker JN, Lawellin DW, Lunt B, McGregor JA: Bacteria and inflammatory cells reduce chorioamniotic membrane integrity and tensile strength. Obstet Gynecol 74:590–596, 1989.

28. Platz-Christensen JJ, Brandberg A, Wiqvist N. Increased prostaglandin concentrations in the cervical mucus of pregnant women with bacterial vaginosis. Prostaglandins 43:133–141, 1992.

29. O'Brien JM, Peeler GH, Pitts DW, Salama MM, Sibai BM, Mercer BM. Cervicovaginal prolactin: A marker for spontaneous preterm delivery. Am J Obstet Gynecol 171:1107–1111, 1994.

30. Darne J, McGarrigle HHG, Lachelin G: Increased saliva estriol to progesterone ratio before idiopathic preterm delivery: A possible predictor of preterm labor? Brit Med J 294:270–272, 1987.

31. Darne J, McGarrigle HHG, Lachelin GCL: Saliva oestriol, oestradiol, oestrone and progesterone levels in pregnancy: Spontaneous labour at term is preceded by a rise in the saliva oestriol:progesterone ratio. Br J Obstet Gynecol 94:227–235, 1987.

32. Maron DJ, McGarrigle HHG, Lachelin G: Lack of normal increase in saliva estriol/progesterone ratio in women with labor induced at 42 weeks gestation. Am J Obstet Gynecol 167:1563–1564, 1992.

33. Clark IM, Morrison JJ, Hackett GA, Powell EK, Cawston TE, Smith SK: Tissue inhibitor of metalloproteinases: Serum levels during pregnancy and labor, term and preterm. Obstet Gynecol 83:532–537, 1994.

34. Brazerol WF, Grover S, Donnenfeld AE: Unexplained elevated maternal serum alpha-fetoprotein levels and perinatal outcome in an urban clinic population. Am J Obstet Gynecol 171:1030–1035, 1994.

35. Williams MA. Hickok DE, Zingheim RW, et al.: Elevated maternal serum alpha-fetoprotein levels and midtrimester placental abnormalities in relation to subsequent adverse pregnancy outcomes. Am J Obstet Gynecol 167:1032–1037, 1992.

36. Milunshy A, Jick SS, Bruell CL, et al.: Predictive values, relative risks, and overall benefits of high and low maternal serum alpha-fetoprotein screening in singleton pregnancies:New epidemiologic data. Am J Obstet Gynecol 161:281–287, 1989.

37. Berkeley AS, Killackey MA. Cedarquist LL: Elevated maternal serum alpha-fetoprotein levels associated with breakdown in fetal-maternal placental barrier. Am J Obstet Gynecol 1146:859–861, 1983.
38. Salafia CM, Silberman L, Herrera NE, Mahoney MJ: Placental pathology at term associated with elevated midtrimester maternal serum alpha-fetoprotein concentration. Am J Obstet Gynecol 158:1064–1066, 1988.
39. Murakawa H, Utumi T, Hasegawa I, Tanaka K, Fuzimori R: Evaluations of preterm delivery by transvaginal ultrasonographic measurement of cervical length. Obstet Gynecol 28:829–832, 1993.
40. Iams JD, Paraskos J, Landon MB, Teteris JN, Johnson FF: Cervical sonography in preterm labor. Obstet Gynecol 84:40–46, 1994.
41. Iams JD, Goldenberg RL, Meis PJ, et al.: The length of the cervix and the risk of spontaneous premature delivery. N Engl J Med 334:567–572, 1997.
42. Rozenberg P, Goffinet F, Malagrida L, et al.: Evaluating the risk of preterm delivery: A comparison of fetal fibronectin and transvaginal ultrasonographic measurement of the cervical length. Am J Obstet Gynecol 176: 196–199, 1997.
43. Jeremias J, Kalo-Klein A, Witkin SS: Individual differences in tumor necrosis factor-α and interleukin-1 production by viable and heat-killed *Candida albicans.* J Med Vet Mycology 29:157–163, 1991.
